# Twenty Years of Entropy Research: A Bibliometric Overview

**DOI:** 10.3390/e21070694

**Published:** 2019-07-15

**Authors:** Weishu Li, Yuxiu Zhao, Qi Wang, Jian Zhou

**Affiliations:** 1 School of Management, Shanghai University, Shanghai 200444, China; 2 College of Sciences, Shanghai University, Shanghai 200444, China

**Keywords:** entropy, bibliometrics, CiteSpace, co-citation, hotspots

## Abstract

*Entropy*, founded in 1999, is an emerging international journal in the field of entropy and information studies. In the year of 2018, the journal enjoyed its 20th anniversary, and therefore, it is quite reasonable and meaningful to conduct a retrospective as its birthday gift. In accordance with *Entropy*’s distinctive name and research area, this paper creatively provides a bibliometric analysis method to not only look back at the vicissitude of the entire entropy topic, but also witness the journal’s growth and influence during this process. Based on 123,063 records extracted from the Web of Science, the work in sequence analyzes publication outputs, high-cited literature, and reference co-citation networks, in the aspects of the topic and the journal, respectively. The results indicate that the topic now has become a tremendous research domain and is still roaring ahead with great potentiality, widely researched by different kinds of disciplines. The most significant hotspots so far are suggested as the theoretical or practical innovation of graph entropy, permutation entropy, and pseudo-additive entropy. Furthermore, with the rapid growth in recent years, *Entropy* has attracted many dominant authors of the topic and experiences a distinctive geographical publication distribution. More importantly, in the midst of the topic, the journal has made enormous contributions to major research areas, particularly being a spear head in the studies of multiscale entropy and permutation entropy.

## 1. Introduction

*Entropy*, making its debut in 1999, is a monthly open access journal, which mainly focuses on the studies of entropy and information. As a member of the Multidisciplinary Digital Publishing Institute (MDPI), it has a full-scale departmental structure and often publishes Special Issues to keep in step with research hotspots. Based on its years of hard work, the journal has gradually arisen as one of the well-known journals in the academic world, indexed by the Science Citation Index Expanded of the Web of Science (WoS) since 2009 and being ranked 22th out of 78 journals in the “Physics, Multidisciplinary” category according to the latest Journal Citation Reports (till the work).

From its inception to the year of 2018, exactly 20 years, *Entropy* had already published 3881 documents, including 3544 articles, 176 reviews, 54 editorials, 18 letters, etc. In particular, 3147 of them are related to the topic of entropy in accordance with WoS criteria, promoting the journal to become a miniature of this distinctive research domain remarkably. Therefore, at this special time, it is quite reasonable and interesting to carry out a retrospective overview in commemoration of *Entropy*’s 20th anniversary.

At present, while there are plenty of disciplines widely used to summarize and analyze the literature, bibliometric methodologies do play an irreplaceable role because of the preciseness and wide applications. According to Broadus [1], bibliometrics is an interdisciplinary study originated in the early Twentieth Century for discovering publications’ development patterns and evolutionary trends from a quantitative perspective, influencing the modern academic world to a great extent.

With decades of development, many commonly-used bibliometric indicators and methods have been gradually carried out and widely accepted, including the quantity of publications, authors, and nations, the number of citations [2], bibliographic coupling [3], journal impact factors [4], co-citation [5], the *h*-index [6], the number of articles above a citation threshold [7], etc. The astonishing growth of math and computer technologies has ushered this discipline into a new era. Researchers in growing numbers started to program visualization software and techniques to enrich and facilitate bibliometric studies, e.g., Pajek [8], Ucinet [9], CiteSpace [10], Histcite [11], Bibexcel [12], VOSviewer [13], etc. Depending on conditions above, numerous articles have been springing up in recent years.

Great numbers of scholars tend to concentrate their studies on core journals. Laengle et al. [14] presented a general overview of the *European Journal of Operational Research* (*EJOR*) for its 40th birthday. Based on the database from WoS, this article first identified *EJOR*’s productive and influential countries, institutions, and authors and then visualized the networks of keywords and journals, respectively, by means of VOSviewer software. Likewise, Cancino et al. [15] provided a bibliometric analysis to celebrate the 40th anniversary of *Computers & Industrial Engineering*. In order to find the development trend of this journal, the study analyzed a series of bibliometric images, like bibliographic coupling, reference co-citation, keywords’ co-occurrence, etc., by VOSviewer. Yu et al. [16] researched the publications of *Information Sciences* from 1968–2016. This work summarized the journal’s attractive and prolific authors, as well as influential documents and synthesized the co-citation network of the references to demonstrate the journal’s research patterns and trends with the help of CiteSpace. Relevant studies also extend to García-Merino et al. [17] for *Technovation*, Cobo et al. [18] for *Knowledge-Based Systems*, Merigó et al. [19] for *International Journal of Intelligent Systems*, Yu et al. [20] for *IEEE Transactions on Fuzzy Systems*, Ji et al. [21] for *Resources Conservation and Recycling*, Yu et al. [22] for *Applied Intelligence*, etc. For more details, please refer to them individually, as their analytical methods and the article structures are almost similar to each other.

There are also plenty of studies focusing on analyzing certain research domains. To some extent, this kind of research is more meaningful and insightful, because it can illustrate the evolutionary process and the hotspots of an area to help readers conduct their research in a scientific way. By using CiteSpace, Chen et al. [23] synthesized reference co-citation networks to recognize important articles related to regenerative medicine, as in those days, this product had penetrated into various kinds of medicine areas at a fast speed. Note that the first author, Chaomei Chen, was the inventor of CiteSpace. Then, as a companion to this article, Chen et al. [24] carried out a bibliometric review about orphan drugs and rare diseases. Fahimnia et al. [25] explored the evolution process, the development trends, and the research interests of the green supply chain by Gephi software. Yu et al. [20] conducted a bibliometric study about Chinese publications on fuzzy theory from 1986–2015. The paper found some patterns and dynamics by sketching co-citation and co-occurrence networks and summarized the information of influential authors and journals, regional distributions of publications, national collaborations, and so on. By means of the new version of CiteSpace, Chen [26] presented a systematic literature review of science mapping. This article is regarded as a milestone of bibliometrics research, analyzing the domain’s structure, dynamic traits, and development trends through co-citation networks. Blanco-Mesa et al. [27,28] demonstrated basic information and development trends in the fields of fuzzy decision making and aggregation operators by using bibliometric indicators and VOSviewer software, respectively. Nunen et al. [29] applied VOSviewer to safety culture to identify its major areas, key journals, prolific nations, collaborative characteristics, etc. For more articles, please refer to [30,31,32,33,34,35,36].

As introduced above, bibliometrics has been improved in steps and is quite helpful in discovering knowledge’s inner patterns and structures, widely used for studying the publications of journals or domains, so that applying it to this article is absolutely rational and irreplaceable. Then, in consideration of the journal’s special name, publication features, and remarkable performance, the work might as well put forward a brand-new bibliometric method to deal with this issue by combining the above two analysis types together. In other words, this study adopts a two-pronged strategy to not only introduce the entire entropy topic, evaluating its publication situations, influential papers, evolutionary path, and hotspots, but also appraise *Entropy*’s influence and characteristics in the meantime.

Setting the word “entropy” as the topic, 123,063 records of publications (only articles and reviews) are directly collected from all the indexes in the WoS Core Collection database or *Entropy*’s official website, in the range of 1999–2018 (from 1 January 1999–31 December 2018, to be more precise). CiteSpace was chosen as the major software for visualization, because, in practice, it is easier to customize and can provide more valuable information than others, widely adopted for bibliometric studies over the world [37].

After this brief introduction as Section 1, Section 2 presents annual publication trends and productive authors and especially uses nation and fund distributions to illustrate geographical differences between the topic and the journal. Section 3 introduces the most cited articles in detail, and journal categories’ information is presented for exemplifying the evolution of research areas relevant to the topic. Then, Section 4 synthesizes the reference co-citation networks to explore not only the evolving process and the hotspots of the entire topic, but also the journal’s status and impacts, followed by Section 5, which summarizes the major conclusions of this study.

## 2. Features of Publication Outputs

In this section, we provide the annual quantity of the topic’s and the journal’s publications and then introduce their most productive authors. Furthermore, according to the results above, the rest of this part uses nation and fund distributions to illustrate *Entropy*’s publishing features.

### 2.1. Annual Distribution of Publications

**Figure 1:**
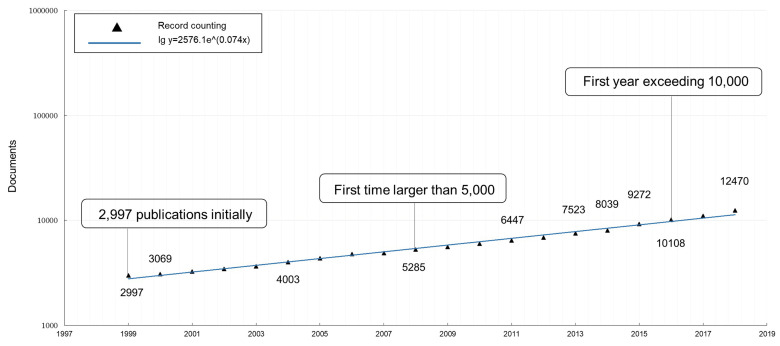
Annual number of the topic’s publications from 1999–2018.

As can be seen in Figure 1, the topic included almost 3000 documents in 1999, and the annual number of publications has enjoyed a continuous increase for the past 20 years. To be specific, the annual quantity has risen from 2997 to 12,470, and especially has been larger than 5000 since 2008 and more than 10,000 since 2016. Nearly half of the articles were published from 2013 onward.

This result is quite significant and meaningful in bibliometrics, because, first, the topic had a tremendous base of publications from the very beginning, so even its modest growth can easily cause a big effect on the scientific world. In addition, according to bibliometric studies, like Price [38,39], in general, the annual publication quantity of an area is growing exponentially over time, rising slowly and then quickly, especially at the early stage of its life, and if the majority of papers are published in recent years, then this research area is considered getting into its vigorous period. By speculation, the topic’s ascent is far from over, which will be astonishingly influencing the science and technology world soon afterwards.

**Figure 2:**
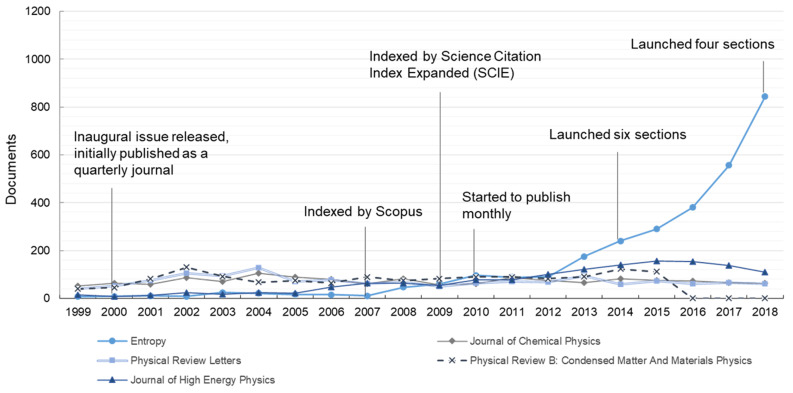
Annual number of the top-five most productive journals’ publications from 1999–2018.

Figure 2 depicts the publishing trends of the top-five most productive journals related to the topic, and particularly marks several historical moments of *Entropy*. The annual publication number of *Entropy* was less than 50 until 2008, while it has dramatically rocketed since 2012, surpassing that of others with great rapidity. However, the other four prestigious, physics-related journals are largely in stagnation or even on the wane in recent years. Combining Figure 2 with Figure 1, the share of *Entropy* in the entire topic outputs has also moved on to a gradual upward arc over the last 20 years, accounting for 0.23% in 1999 and for 6.77% in 2018. All the information not only demonstrates the fast growth of *Entropy*, but also indicates the development and expansion of the entire topic.

### 2.2. Most Productive Authors

As an important part in traditional bibliometric analysis, productive authors are considered to be introduced in detail, because they are the major dedicators and may even lead the directions of their domains.

Table 1 lists the top-20 prolific authors of the topic in the descending order of publication quantity. Indicators from left to right are rank, name, institution, country or region, total publications (TP), total citations (TC), total citations per publication (TC/TP), *h*-index, and citation thresholds. The nations are judged by the locations of institutions written in the documents, not the authors’ real nationality.

Apparently, most of the institutions are located in Asia, America, and Europe. Over half of the authors are Chinese, and four of them are working at the Chinese Academy of Sciences, a linchpin for researching high technology and natural sciences, suggesting that Chinese scholars have played a vital role in this domain.

The top-three authors are Lingen Chen, Fengrui Sun, and Angelo Plastino. Chen and Sun are both professors at People’s Liberation Army of China (PLA) Naval University of Engineering. Chen specializes in energy and power engineering and modern thermodynamics, and Sun is an expert in energy and power engineering and engineering thermophysics. Due to their working relationship and similar research domains, the two have collaborated in research for a long period of time, producing a great number of articles relevant to the entropy topic. For example, in Table 1, Chen and Sun shared a paper [40] having more than 500 citations together. This paper forecasted the future direction of finite thermodynamics by reviewing the study’s historical background, research development, and theories. The third author is Angelo Plastino, an emeritus professor and physicist at National University La Plata. He is mainly interested in information theory, statistical mechanics, and quantum information, showered with innumerable honors and prizes.

Moreover, in Table 1, a paper authored by Jienwei Yeh [41] has been cited more than 2000 times. This paper, a landmark in materials science and engineering, provided a new method for designing nanostructured high-entropy alloys. Jienwei Yeh is a professor working at National Tsing Hua University in Taiwan, China, having considerable findings on materials science, especially high-entropy alloys.

The most productive authors of *Entropy* are listed in Table 2. Obviously, the two tables contrast sharply with each other. In the first place, there are some familiar figures appearing again, like Lingen Chen, Angelo Plastino, and Vijay P. Singh, both in Table 1 and Table 2. Besides, the countries and institutions listed in Table 2 are more plentiful than in Table 1, which indicates that the journal has a distinctive geographical distribution. This phenomenon is meaningful and is worth being further investigated because as discussed by Liang and Zhu [42], it might illustrate the spatial differences of publication quantities and cooperation. Thus, the work visualizes nation and fund distribution networks to deal with this issue in the latter part of this section.

The top-three prolific authors of the journal are Dumitru Baleanu, Vijay P. Singh, and Angelo Plastino, from Turkey, the USA, and Argentina, respectively. Dumitru Baleanu is a professor interested in fractional dynamics and its applications, fractional differential equations, mathematical physics, and so on. He is productive in various fields, writing or participating in over 200 articles. Vijay P. Singh is a distinguished hydrologist at Texas A&M University, specialized in biological and agricultural engineering with plenty of honors and awards. His current interests include surface-water hydrology, groundwater hydrology, hydraulic engineering, irrigation engineering, etc.

The most attractive author in Table 2 is Yudong Zhang, who has two articles receiving more than 100 citations, respectively. Zhang is a professor now working at University of Leicester, mainly focusing on knowledge discovery and machine learning. As for the two papers, the first one [43], honored as a highly-cited paper by WoS, proposed a new automatic system of computer-aided diagnosis, which is more accurate for magnetic resonance brain images, and the other [44] presented a new approach for image segmentation by creatively employing Tsallis entropy rather than Shannon entropy.

To sum up, there are plenty of researchers devoting themselves to the entropy topic, especially to physics-related areas, which has made tremendous impacts on the scientific world. *Entropy* has attracted large numbers of celebrities and key scholars in different areas and has gained acceptance worldwide. Nevertheless, on the one hand, *Entropy*’s citation situation is relatively weak, which will be further discussed when talking about the most cited papers in Section 3. On the other, through investigation, or it also can be partly told from the content above, the topic’s research areas seem a little different to that of the journal, as if they have diverse taste and interests for publications. This difference will be explored and studied in Section 4, since data here could not provide an overall landscape.

### 2.3. Nation Distribution Analysis

The nation relationships of the topic and the journal are portrayed by CiteSpace. According to CiteSpace textbooks, a node represents a country, and its radius is in proportion to the country’s publication quantity. A line linking two nodes symbolizes the cooperation between the two countries. A country is considered to play a pivotal role in cooperation if its node is surrounded by a purple ring. Colors reflect the chronological order by changing from dark to light. Specifically, the different colors of a node indicate the country’s different publication years, and a line’s color presents the first year that the two countries cooperated with each other. Due to CiteSpace’s design, not all of the articles can be identified and visualized, so in this article, we selected the top-300 and the top-100 most cited papers in each year for the topic’s and the journal’s visualization, respectively.

**Figure 3:**
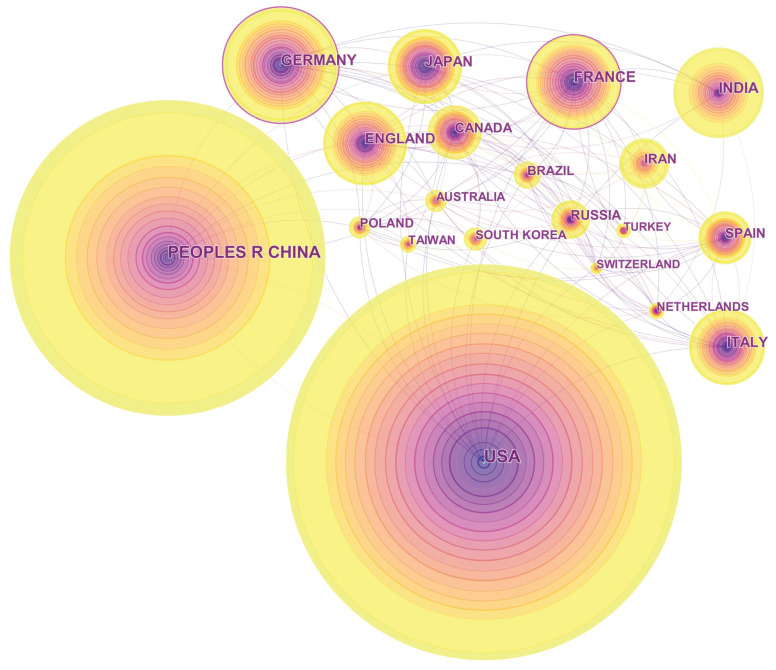
The network of the top-20 most productive countries or regions of the topic from 1999–2018.

Figure 3 displays the top-20 productive countries or regions of the topic. Most of them are in Europe (9), Asia (8), and North America (2). The USA and China are the top-two prolific countries in history and have still maintained their high productivity of late in terms of their thick, light tree-rings. Germany ranks in third place, followed by France, India, England, and Italy, successively. Iran, Brazil, India, and Russia have published plenty of literature in recent years, while the speed of Japan, Spain, Italy, and Canada is slowing down to some extent, which points to the fact that developing countries are growing more quickly by comparison. This phenomenon also appeared in the analysis of *EJOR* [14].

Surprisingly, the nodes of the USA and China do not have purple rings, whereas those of Germany and France do. Three reasons can mainly explain this result. To begin with, there are numerous researchers and institutes in China and the USA, so that it is easy for researchers in both countries to find domestic partners working on the same topic. Comparatively, scholars in small- or medium-sized countries are more likely to seek cooperation internationally. Secondly, the entropy topic has already penetrated into many disciplines, especially physics and engineering, which are traditional and powerful domains in Germany and France. As a result, the two countries could easily win popularity in international cooperation. At last, through further investigation, the majority of countries contributing to the topic are in Europe. Therefore, the influence exerted by Germany and France might be relatively strong and durable.

**Figure 4:**
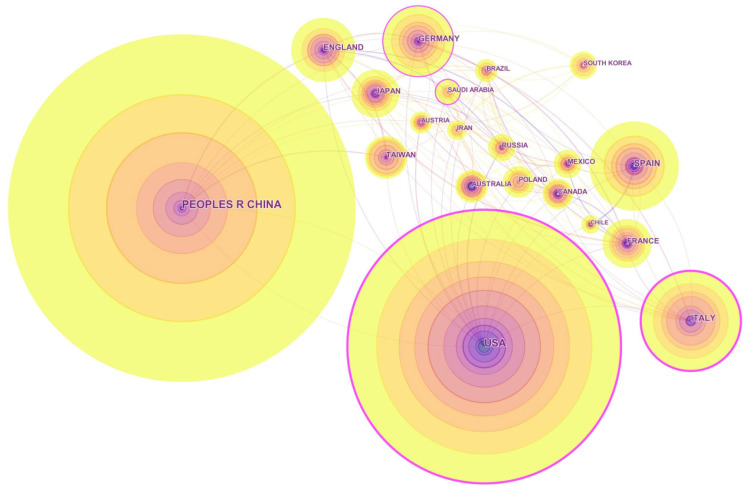
The network of the top-20 most productive countries or regions for *Entropy* from 1999–2018.

Figure 4 shows the top-20 productive countries or regions of the journal. Apparently, there are many differences between the two figures. First, countries or regions in Figure 4 are in Europe (8), Asia (6), South America (3), North America (2), and Australia (1), which compose a greater geographic scope. Second, China has replaced the USA, becoming the most productive country, and the countries from the third to the fifth are displaced by Italy, Spain, and Germany. Note that Japan and Spain are active in the journal, while they are both on the decline in the entire topic. Taiwan, a region ranking 19th out of 20 and almost invisible in Figure 3, has climbed to the tenth position in Figure 4. All the evidence suggests that *Entropy* is particularly attractive among Asian scholars.

Furthermore, Saudi Arabia has a purple ring in Figure 4, which is somewhat unexpected because of its late start in education and research. By survey, in *Entropy*, there are in total 88 papers (including but not limited to the topic) authored by Saudi Arabia from 1999–2018; the first one was in 2011, and 79 of them were written by international collaboration, enjoying near the highest collaborative rate among major countries. Unlike other countries preferring to team up with developed countries, Saudi Arabia is more willing to cooperate with its neighbors, like China, Pakistan, Turkey, Romania, Iran, etc. Presumably in recent years, Saudi Arabia tried to employ or collaborate with foreign researchers in an effort to enhance its research reputation. Generally, such high-level international cooperation can inflate research development rapidly, but may also cause severe problems in its scientific infrastructure, which should be a concern.

Despite all this, the result still indicates that major research communities in *Entropy* are around Germany, Italy, the USA, and Saudi Arabia, located in Europe, North America, and the Middle East, respectively. China, however, is still without a purple ring. Although the country has numerous publications and plays a key role in this area, its international cooperation needs to be improved as soon as possible for further development.

### 2.4. Fund Distribution Analysis

In order to deal with this issue, we first survey the topic’s funds. There are over 50,000 funds participating in the topic, mainly located in Europe, North America, and China, and the top-20 are largely governed by Europe (5), the USA (5), China (4), and Canada (2). The National Natural Science Foundation of China (NSFC) is the dominant one, publishing more than 12,000 documents during the last 20 years, followed by the National Science Foundation (NSF, USA, 4910), the Fundamental Research Funds for the Central Universities (China, 1437), the Engineering and Physical Sciences Research Council (U.K., 1322), and the National Institutes of Health (NIH, USA, 1143), in descending order by publications.

**Figure 5:**
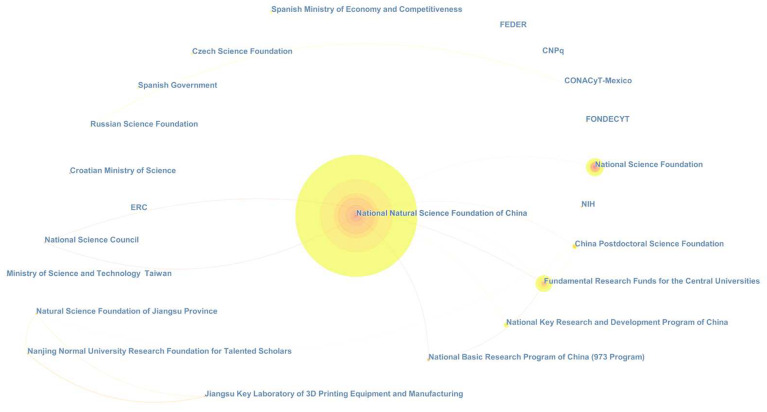
The network of the top-20 most productive foundations of *Entropy* from 1999–2018 (including ties).

The major funding agencies of the topic and the journal are quite similar to each other, mainly including natural science foundations granted by governments at national or ministerial levels, whereas the biggest difference is that in *Entropy*, there are many smaller, regional Chinese funds emerging at the top of the rank. To be specific, there are in total 1922 foundations supporting the studies in *Entropy*, and the majority of them are established by China (32.62%), the USA (22.94%), Spain (12.33%), Germany (7.28%), and Italy (5.57%). Concerning that most of the funds cannot be visualized due to the great mismatch of publication quantity, we only depict the top-20 most productive foundations of *Entropy* by CiteSpace in Figure 5. In the figure, a node represents a foundation, and other basic instructions are the same as in Section 2.3.

As can be seen, all the funds are in China (10), Europe (7), South America (3), and the USA (2). Funds in Europe and the Americas are at the upper right and left, respectively. There are two USA foundations, i.e., NSF and NIH. Spain is the most active country in Europe, owning two foundations in the figure.

Nearly half of the foundations are organized by Chinese administrations, occupying the entire lower part of the depiction. Still, NSFC is the most prolific one, which acts as a tremendous hub among Chinese funds and is even the key to push *Entropy* moving forward. Small-sized Chinese foundations include the Ministry of Science and Technology Taiwan, the Nanjing Normal University Research Foundation for Talented Scholars, the Jiangsu Key Laboratory of 3D Printing Equipment and Manufacturing, etc., which are managed by local governments and schools.

This difference may be principally ascribed to Chinese research policies and open access journals’ business method. In China, the promotion and the payment of researchers, especially those who are young and have no place in the academic world, are highly relevant to their number of papers per year. For them, small funds are easy apply for, and open access journals’ quality and publishing speed can meet their needs appropriately, even though they have to pay some money. With this in mind, it also can be imagined that Chinese researchers will play a more and more important role in open access journals as Chinese local academies and foundations have been increasing rapidly in recent years.

## 3. Most Cited Publications

Papers receiving more citations than others may contain the imperative or fundamental knowledge of their domains, which needs to be carefully studied. Therefore, Section 3 is arranged in order to find out the topic’s and the journal’s most attractive documents and to catch a glimpse of the major research areas.

### 3.1. Most Cited Literature of the Topic

Table 3 presents the top-20 most cited documents of the topic in descending order of their citations from 1999–2018. The work nearly provides all of the commonly-used indicators, i.e., title, author, publication year, journal’s name (Journal), journal’s category (Category), total citations (TC), total citations per year (TC/Year), number of authors (NA), number of institutions (NI), and number of references (NR), in order to provide full-scale information from different perspectives.

At first, two facts should be explained. The mean publication year was about 2003, while this result is quite reliable because articles always need a long time to be widely accepted, and 10 more years is a must to accumulate sufficient citations, according to bibliometrics research [45]. In addition, more than a third of the papers in the list are reviews. Actually, this is natural and reasonable since high-quality reviews can provide comprehensive information and research tendencies, which are extremely useful to scholars.

The first article [46], a milestone astonishingly gaining more than 10,000 citations, provided an abstract framework and an information operator for the research of compressed sensing of objects. Its author, David L. Donoho, is a professor of statistics and of humanities and sciences at Stanford University. As a mathematician, David L. Donoho has immensely contributed to statistics, signal processing, and harmonic analysis, especially his algorithms, which have considerably promoted the maximum entropy principle. The second article [47] used the maximum entropy method in species geographic distributions because of its simplicity and accuracy. The third one [48], also a proceedings paper, proposed the holographic principle, a breakthrough in physics. Juan Maldacena, the author, is a theoretical physicist working at the Institute for Advanced Study in America, famous for his research on the holographic principle. Then, through further studies, many articles and scholars of the topic are highly related to physics, ecology, and computer science. Therefore, the work might as well list the most attractive papers in recent years for reference, since these areas are growing or upgrading at a fast rate.

Table 4 lists the topic’s top-20 attractive documents over the last five years. In contrast, there are more reviews appearing in Table 4, which demonstrates that this publication type is becoming more and more popular and in demand with time. Furthermore, the average values of NA, NI, and NR of Table 4 are much higher than those of Table 3, regardless of the first article [49], which was written by a collaboration including 244 authors and 99 institutions. Usually, an article authored by a large number of authors, institutions, as well as countries is considered to be more complex, difficult, and extensive, and it might be profound and detailed when referring to plenty of references.

Nevertheless, the most intriguing thing might be that the categories in Table 4 are more diverse than those in Table 3, and multidisciplinary, materials, and physics do appear more frequently in the former. In bibliometrics, this phenomenon may exemplify the evolution of research trends, which is worthy of an in-depth analysis.

**Figure 6:**
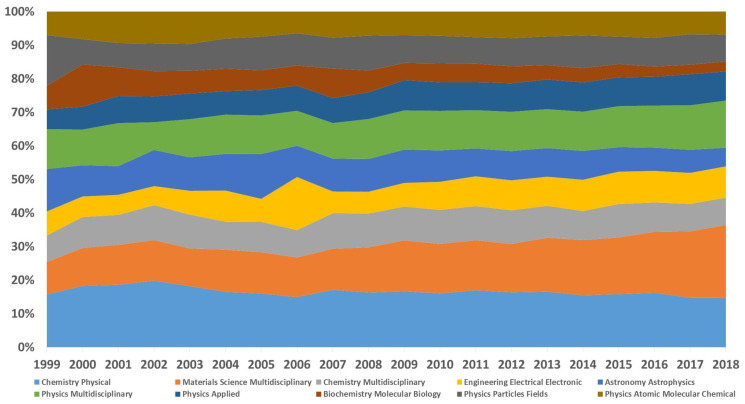
The proportion change of the categories of the topic from 1999–2018.

Firstly, from a quantity perspective, Figure 6 portrays the proportion change of the top-10 categories relevant to the topic with a 20-year timespan. Apparently, the categories have not yet changed from beginning to end, while their proportions have varied in part. Over the past two decades, materials science multidisciplinary is the fastest growing category, which has risen by about 12%, followed by physics applied (2.8%), physics multidisciplinary (2.2%), and engineering electrical electronic (2.1%), yet the shares of astronomy astrophysics, physics particles, and biochemistry molecular biology have declined by more than 7%, 7%, and 4%, respectively. Furthermore, we investigated the first year that each category appeared in the topic and found several typical time intervals. For example, physics- and math-related categories appeared frequently for the entire 20 years. Categories focusing on chemistry mainly emerged in 2000. Social sciences categories intensively surfaced in the range of 2006 and 2008. Multidisciplinary studies categories and natural sciences categories have constantly exploded since 2007 and 2009, respectively.

Even though these depictions are relatively rough and abstract, they still indicate that the change of research areas really exists. Combined with the information in Section 2, this analysis also explains the reason why *Entropy* has published quickly since the last seven years. By survey, *Entropy* has conducted a series of reforms and innovations in an effort to deal with the boom of various kinds of entropy articles. For example, professor and doctor Kevin H. Knuth has taken over as Editor-in-Chief since 2012; six sections were launched in 2014; and four new sections started to operate in 2018. All these improvements diversified its publications and improved its ability to better accommodate the change of the topic.

In short, all the evidence discloses the fact that entropy research is becoming more and more extensive, sophisticated, and interdisciplinary, so that research cooperation should be valued and emphasized unprecedentedly, and how to get general and specific research trends in a scientific way has increasingly become a key issue for domain experts.

### 3.2. Most Cited Literature of Entropy

The top-20 most influential documents in *Entropy* are listed in Table 5. There are three articles gaining over 200 citations. The most cited one [50] was published in 2014 by six authors from the Research Laboratory governed by the U.S. Air Force, proposing a high-entropy alloys design and evaluation method, which is mainly applied to transportation and energy industries. The second article [51] celebrated the 10th anniversary of permutation entropy by summarizing its theoretical foundations and major applications in areas of economical markets and biomedicine. The third paper [52] is a review that held that maximum entropy can be useful in recognizing or distinguishing wild animal’s distributions and habitat selection and introduced the model’s advantages, disadvantages, as well as future improvements in great detail. This review cited many references, like [47], the second most cited article of the topic over time, and can be considered as an overall retrospect about relevant research. These three articles above are all labeled as highly-cited papers by WoS. Then, through further survey, the journal has inherited and improved several areas of the topic and also has its own research favorites, which will be vividly demonstrated in Section 4.

Moreover, three facts should be explained. Above all, three quarters of the journal’s documents are published in Special Issues. This well-targeted publication method could help researchers to find their interests in a more rapid and precise way, reflecting that the journal has been successful in its Special Issue development and really has a keen eye for research hotspots. Next, the average values of publication years, NA, NI, and NR in Table 5 are all larger than those in Table 3. As mentioned above, higher values of these indicators are often coupled with better performance. Although this can be partly ascribed to *Entropy*’s publication burst in recent years, the fact still displays the journal’s gradual improvement. Thirdly, it cannot be denied that there is a large gap between *Entropy* and prestigious journals, as no article of *Entropy* ranks on Table 3 and Table 4. This conclusion seems to contradict *Entropy*’s good performance and high impact factor.

After a comprehensive survey, two reasons can largely account for this result. First, the performance of *Entropy* can be attributed to not only its continuous advancement, but also open access journals’ publication policies. Papers submitted to these journals would be quickly reviewed, and everyone has the equal right to read and cite them freely when they are accepted and published online, which has contributed to *Entropy*’s high popularity. Second, highly-cited papers on the topic (including, but not limited to publications in Table 3 and Table 4) are always related to theoretical and practical breakouts, while *Entropy* likes to issue reviews or articles that are easy to read and not so technical. Honestly, researchers in large numbers are more willing to publish their masterpieces in journals that are time-tested and technical-oriented, even though they may wait for a long time. Therefore, in such cases, *Entropy* is not their best choice.

## 4. Reference Co-Citation Networks

Along with the analyses above, the work not only witnesses the achievements of the topic and the journal, but also finds some evidence that indicates the topic’s evolution and *Entropy*’s publication interests. As a consequence, this section is aimed to further investigate these phenomena and to explore the hotspots by using reference co-citation networks.

Co-citation, proposed by Henry Small [5] in 1973, is a commonly-used measure in bibliometric research, appearing when two articles are cited together by any other papers. Conspicuously, the more citations two articles obtain together, the more related they should be. Over time, these relationships would be gradually synthesized as a huge network, which can vividly symbolize publications’ evolutionary process, research areas, and hotspots.

### 4.1. Co-Citation Networks of the Topic

**Figure 7:**
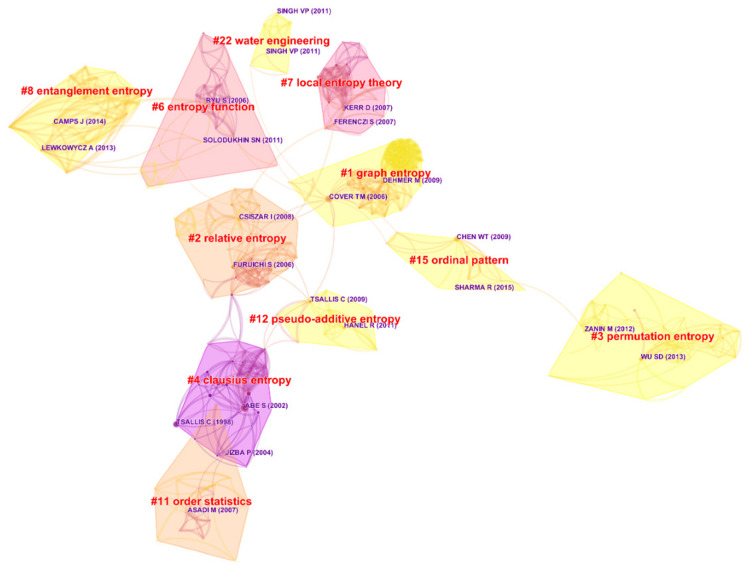
The reference co-citation network of the topic from 1999–2018.

Visualized by CiteSpace, Figure 7 presents the co-citation network of the topic from 1999–2018, depicting 11 clusters, which are more stable and significant. According to Table 6, these clusters account for more than 42.13% of the entire references, from which the panorama of the topic could be fairly displayed.

Technical instructions should be introduced at first. In co-citation networks, clusters are ranked by their sizes, and their labels are keywords extracted from the references by log-likelihood ratio (LLR) algorithm. For completeness, the work also labels the clusters by two other famous algorithms, i.e., term frequency-inverse document frequency (TF-IDF) and mutual information tests (MI). A node, representing a reference, is colored in deep red if it has a citation burst, which reflects that the reference was significantly active at one point in history. Furthermore, it should have a dark purple legend composed of the author’s name and publication year since the reference is highly cited in its cluster over time. A line linking two nodes symbolizes the two papers’ co-citation relationship, and its thickness is in proportion to the frequency that the two paper have been co-cited. Colors reveal the chronological order, similar to the criteria listed in Section 2.

Cluster #1, for example, is the largest one unanimously named as graph entropy by the three algorithms. It has a 0.992 silhouette score in Table 6. The silhouette score reflects a cluster’s good homogeneity or consistency when it tend to close to one, and a 0.992 silhouette score is usually considered extremely high.

References highly cited or with citation bursts in Cluster #1 include [53,54,55,56], publishing in 2006, 2009, 2011, and 2008, respectively. The first one [53] is a fundamental textbook named *Elements of Information Theory*, which introduced nearly all of the essential knowledge in information theory. Needless to say, its author, Thomas M. Cover, is a great information theorist and past president of the IEEE Information Theory Society, dedicating his entire life to promoting the mix of information theory and statistics. Subsequently, the second paper [54] analyzed entropy-based molecular descriptors in chemical use. The third document [55] offered an overall introduction about the measures of graph entropy from a historical view, and the fourth article [56] provided a general structure of graph entropy and explored relationships among several kinds of graph entropies. The articles from the second to the fourth are all authored by Matthias Dehmer, a professor now teaching at the University of Applied Sciences Upper Austria, who has plenty of interests like data science, bioinformatics, machine learning, information theory, computational statistics, etc. In simple terms, Cluster #1 is a big family about entropy, graph entropy, and information theory, as well as their applications, reflecting entropy’s, especially graph entropy’s, development and practicability.

Then, we further investigate the most cited papers of Cluster #1 to explore the hotspots of this cluster, as in an area, citing papers is always the latest or representative extension of cited papers. These papers include [57,58,59,60,61], etc., mainly talking about new types of graph entropy measures and their extremal properties. Apparently, the research of Cluster #1 is still at the initial stage, i.e., theory development, and how to design graph entropy measures and prove their extremal values are the principal works at present.

**Figure 8:**
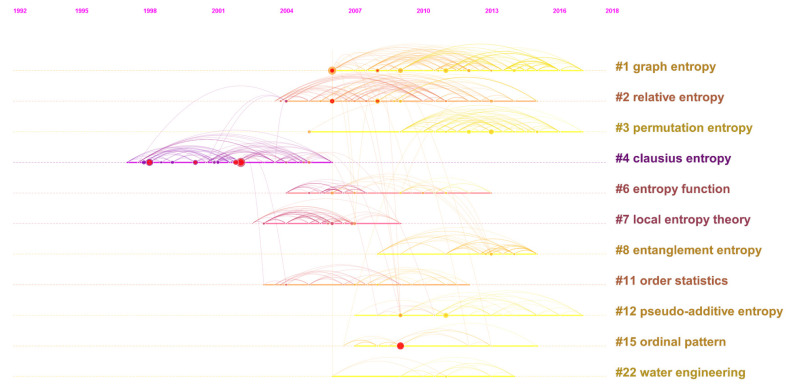
The reference co-citation network of the topic by timeline from 1999–2018.

Figure 8 displays the relationships among the clusters in terms of timeline to make up the simplistic structure of Figure 7. As can be seen, Cluster #4, labeled as Clausius entropy, composability, and *q*-exponential distribution, is the oldest one existing from 1997–2006, which includes many papers having citation bursts. Therefore, by speculation, this cluster had declined and almost perished years ago after previous prosperities. Nevertheless, Cluster #4 closely connects with the sources of Clusters #2, #11, and #12, suggesting that it might largely affect these clusters or be the knowledge base of them. According to their life spans, the bars of Clusters #6, #7, and #11 are in dark color, relatively short, and without citation bursts, and even do not last to the present, which illustrates that these clusters are outdated and short of research value. However, Clusters #6 and #8 are highly related, so presumably Cluster #8 has carried forward the studies of Cluster #6.

Definitely, the most important messages in Figure 8 are that Clusters #1, #3, and #12 still continued their strong performance in recent years, which should be surveyed in great detail because they may still maintain their tendencies currently.

Cluster #3 can be titled as permutation entropy, multiscale entropy, or detecting weak abrupt information, enjoying the longest lifetime from 2005–2017. Its papers that are highly cited or with citation bursts include [51,62,63]. The first paper [62] is a review presenting the theoretical bases and major applications of permutation entropy. The second one [51] provided a concept named composite multiscale entropy, which is more suitable for practical use because it has overcome the handicap of traditional multiscale entropy. The third article [63] applied the multiscale entropy method to human heartbeat fluctuations in order to verify the method’s capability for biological signals’ measurement. In short, this cluster basically refers to multiscale entropy, permutation entropy, and their applications. Wu [62] and Zanin [51] were issued by *Entropy*.

The most cited papers of Cluster #3 include [64,65,66,67], etc. These papers referred to theoretical and practical reviews of entropy methods [64] and permutation entropy [65], new entropy types based on permutation entropy [66], and a new algorithm for accelerating entropy computation [67], respectively. Unlike Cluster #1, Cluster #3 is relatively mature, and now, its theoretical innovation and optimization might be worthy of attention.

Additionally, in Figure 7, Zanin [51] is strongly co-cited with Sharma [68], a paper in Cluster #15. By survey, Sharma [68] employed vast numbers of entropy-based algorithms for electroencephalogram (EEG) signals’ evaluation, and Cluster #15, known as ordinal pattern, sample entropy, and multiscale permutation, can be considered as the practical extension of Cluster #3. To be specific, the milestones in Cluster #15 include [68,69,70,71,72], mainly discussing the applications of permutation entropy, especially in clinical and medical use. Note that Sharma [68] and Unakafova [70] were also published by *Entropy*, which demonstrates the journal’s superiority and acceptance in this field.

Cluster #12 is titled as pseudo-additive entropy, Tsallis entropy, and different entropy formalism, respectively. This cluster mainly talks about Tsallis entropy, Tsallis statistics, and their applications, including some foundational articles like [73,74,75], etc. The first paper [73] provided some important and basic results about Tsallis entropy. Its author was Constantino Tsallis, a well-known theoretical physicist and also the great contributor of Tsallis entropy and Tsallis statistics. In the second article [74], the author employed Tsallis entropy and Kaniadakis entropy to build up a minimal entropy martingale for semi-Markov regime switching interest rate models, and the third reference [75] classified entropies according to their asymptotic scaling.

The most cited papers to Cluster #12 include [76,77,78,79,80], etc. Except a review about entropy application in the fields of mathematics and science [80], other papers mainly introduced the theoretical innovation or extension of different kinds of entropies, especially those that are related to entropy functionals [76,78]. It seems that Cluster #12 is sophisticated and has integrated with other entropy studies, so that research across different entropy areas and ensuing application may have potential for research.

### 4.2. Co-Citation Networks of Entropy

**Figure 9:**
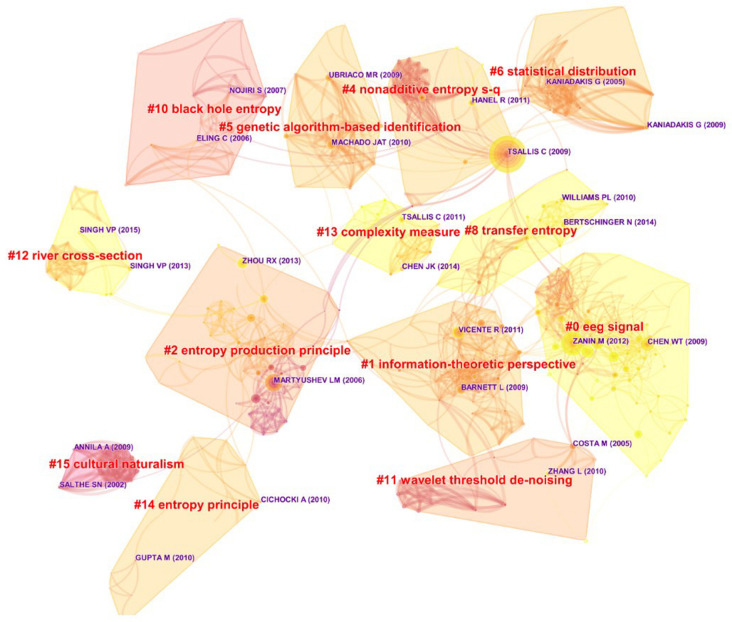
The reference co-citation network of *Entropy* from 1999–2018.

**Figure 10:**
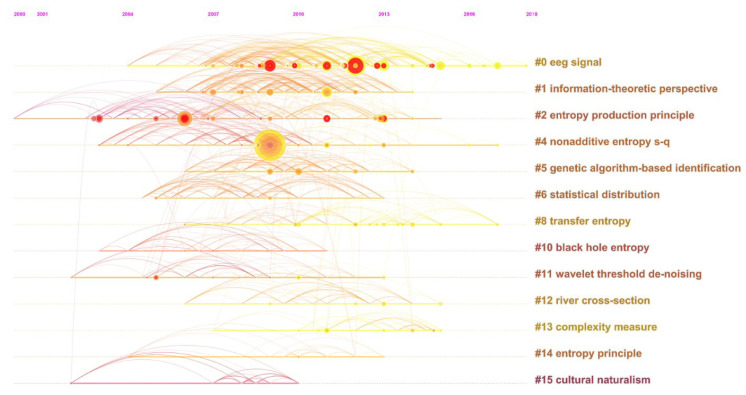
The reference co-citation network of *Entropy* by timeline from 1999–2018.

Similarly, Table 7 and Figure 9 and Figure 10 present the co-citation information of *Entropy*, including 13 clusters.

The most superficial phenomenon is that in Table 7, the labels of each cluster are more identical, suggesting that *Entropy*’s clustering result is more stable and clearer. However, in Figure 10, nodes and lines are intertwined almost in a crisscross pattern, so that it seems that the journal’s clusters are significantly related, neck and neck with each other, and difficult to distinguish.

Several reasons can account for this paradox: Firstly, *Entropy* has 12 independent publishing sections. Therefore, its research areas would grow up side by side, and corresponding references can be synthesized in an accurate way. secondly, as an important part of the journal, interdisciplinary publications unavoidably need to refer to different kinds of disciplines, which contributes to the complexity of co-citation relationships; thirdly, an entire topic is even more complicated than a journal belonging to it, thus the topic’s labels are more difficult to summarize; and at last, as mentioned before, CiteSpace has a bunch of data sifting and slicing criteria, so that not all of the nodes and lines can be seen when visualized, which makes the topic’s networks relatively simple. As a consequence, this phenomenon is really possible to occur and coincidentally reveals *Entropy*’s interdisciplinary nature.

By comparison, some clusters between Table 6 and Table 7 are exactly the same, like maximum entropy and black holes, if all kinds of labels can be employed. Furthermore, it can be seen that *Entropy* has some distinct areas, including transfer entropy, discrete wavelet entropy, etc. This result seemingly reflects the contribution and the innovation of the journal in the midst of the topic. However, perhaps affected by publication quantity, *Entropy*’s labels are more related to certain specified theory extensions or applications, while those of the topic are highly relevant to the basic concepts of entropy. Therefore, in this way, the role *Entropy* played can only be partly demonstrated, and more detailed analysis still needs to be conducted.

Here, we use Cluster #0 to further discover *Entropy*’s potential influence. Described as EEG signal or fault diagnosis, Cluster #0 has the most articles with citation bursts, including [51,62,68,72,81,82,83,84,85] and enjoys nearly the longest lifespan from 2004–2018. Briefly speaking, Cluster #0 refers to the theoretical and practical extensions of multiscale entropy and permutation entropy, especially involving clinical medicine and EEG signal applications. As introduced before, the works of [51,62,68,72] in *Entropy*’s Cluster #0 are also the mainstays of the topic’s Cluster #15 or #3, and the works of [51,62,68] were published by *Entropy*. All messages indicate that *Entropy*’s Cluster #0 plays a dominant role in the studies of multiscale entropy and permutation entropy, especially in EEG signal application.

In summary, during the last 20 years, the mainstream research has changed remarkably, and still, there are several strong and active domains meriting swiftly being studied and followed. Besides, the contrastive analysis reveals that *Entropy* not only puts forward some distinctive research areas, but also has played a significant role in several cutting-edge areas of the topic.

## 5. Conclusions

In 2018, *Entropy* enjoyed its 20th birthday, so that the work intended to provide a bibliometric overview in commemoration of its anniversary.

Through document investigation, the work proposed a new bibliometric analysis method to respond to the journal’s features by analyzing the topic and the journal together. Based on the data from WoS in the range of 1999–2018, this review successively introduced the entropy topic’s publication situations, influential papers, evolutionary path, and hotspots, and in this context, *Entropy*’s impacts and inner patterns have been uncovered step by step. Major conclusions and comparisons are listed in Table 8.

In short, the entropy topic has already influenced or penetrated into various kinds of disciplines, showing its muscles and great potentiality to the academic world. According to the geographical distributions, the USA and China are the top-two productive nations, whereas European countries, especially Germany and France, play a pivotal role in international cooperation. The majority of the funds supporting the topic are natural science foundations constructed by European, North American, and Chinese governments at national or ministerial levels. Through decades of evolution, the research areas have varied significantly, and the hotspots mainly include graph entropy, permutation entropy, pseudo-additive entropy, etc. Specifically, for graph entropy, the key work is to improve its theory structure; for permutation entropy, theoretical innovation and optimization matter greatly; and for pseudo-additive entropy, cross-over study and application might be the first priority.

Besides, *Entropy* has experienced an astonishing growth in recent years, attracting large numbers of the scholars who are indispensable in the topic. By comparison, *Entropy* is more popular among Asian researchers, and its major cooperation communities are located in Europe, North America, and the Middle East, which are more diversified and international. The foundation situation of *Entropy* resembles that of the topic, but the journal is more preferred by smaller, regional foundations in China. Moreover, with respect to research domains, the journal has contributed much to the topic, especially leading the trends of multiscale entropy and permutation entropy.

## Figures and Tables

**Table 1:** The top-20 most productive authors of the topic from 1999–2018.

Rank	Author	Institution	Country or Region	TP	TC	TC/TP	*h*	>2000	>1000	>500	>200	>100	>50	>20	>10
1	Chen LG	Naval Univ Engn	China	257	5397	23.10	40			1	1	3	24	98	165
2	Sun FR	Naval Univ Engn	China	222	5194	23.40	38			1	1	1	19	91	152
3	Plastino A	La Plata Natl Univ	Argentina	211	3470	16.45	32				1	5	14	46	90
4	Shen BG	Chinese Acad Sci	China	190	5840	30.74	35			1	5	8	21	69	112
5	Sun JR	Chinese Acad Sci	China	170	5507	32.39	33			1	5	8	19	63	105
6	Hlil EK	Univ J Fourier	France	166	1631	9.83	21						1	23	61
7	Kumar A	Indian Inst Technol	India	157	1886	12.01	25					2	6	27	46
8	Tan ZC	Chinese Acad Sci	China	153	1358	8.88	18						2	17	38
9	Yeh JW	Natl Tsing Hua Univ	Taiwan	144	11,589	80.48	52	1	1	1	15	30	53	95	111
10	Veldhuis JD	Mayo Clin	USA	144	4235	29.41	37					7	24	72	109
11	Smirnova NN	Natl Res Lobachevsky State Univ	Russia	144	700	4.86	13							2	18
12	Singh VP	Texas A&M Univ	USA	139	1969	14.17	24						5	35	69
13	Obada ASF	Al Azher Univ	Egypt	131	1188	9.07	19							19	46
14	Zhang Y	Univ Sci & Technol Beijing	China	129	5693	44.13	35		1	1	4	10	21	58	75
15	Yu SC	Chungbuk Natl Univ	South Korea	128	2799	21.87	24		1	1	1	4	11	28	44
16	Liaw PK	Univ Tennessee	USA	127	5331	41.98	32		1	2	4	8	19	51	68
17	Du YW	Nanjing Univ	China	121	3379	27.93	31			1	1	5	13	47	75
18	Hu FX	Chinese Acad Sci	China	119	4456	37.45	29			1	5	7	15	43	71
19	Kumar GS	CSIR Indian Inst Chem Biol	India	119	2756	23.16	29					4	13	50	81
20	Cheikhrouhou A	Digital Res Ctr Sfax	Tunisia	118	1337	11.33	22						3	26	44

Abbreviations: TP: total publications; TC: total citations; TC/TP: total citations per publication; *h*: *h*-index; >2000, >1000, >500, >200, >100, >50, >20, and >10: Number of papers with more than 2000, 1000, 500, 200, 100, 50, 20, and 10 citations.

**Table 2:** The top-20 most productive authors of *Entropy* from 1999–2018.

Rank	Author	Institution	Country or Region	TP	TC	TC/TP	*h*	>100	>50	>20	>10	>5
1	Baleanu D	Cankaya Univ	Turkey	17	168	9.88	7			2	7	10
2	Singh VP	Texas A&M Univ	USA	17	95	5.59	5			1	4	5
3	Plastino A	La Plata Natl Univ	Argentina	16	77	4.81	5				3	5
4	Machado JAT	Polytechnic Institute Of Porto	Portugal	14	127	9.07	5			3	5	5
5	Lopes AM	Univ Porto	Portugal	12	93	7.75	4			2	3	3
6	Feidt M	Univ Lorraine	France	11	74	6.73	4			1	1	3
7	Isaacson LK	Univ Utah	USA	11	33	3	4					3
8	Rashidt MM	Univ Birmingham	England	10	143	14.3	7		1	3	4	8
9	Principe JC	Univ Florida	USA	10	64	6.4	5				4	6
10	Zhang YD	Univ Leicester	England	9	509	56.56	8	2	4	6	8	9
11	Ay N	Max Planck Inst Math Sci	Germany	9	126	14	5		1	1	5	6
12	Chen LG	Naval Univ Engn	China	9	95	10.56	4		1	2	2	3
13	Seneff S	MIT	United States	8	162	18	6		1	2	5	8
14	Porta A	Univ Milan	Italy	8	121	15.13	4		1	2	3	4
15	Livadiotis G	Southwest Res Inst	United States	8	76	9.5	4			3	3	4
16	Ibrahim RW	Univ Malaya	Malaysia	8	57	7.13	4				3	4
17	Barranco-Jimenez MA	Inst Politecn Nacl	Mexico	8	45	5.63	4				2	3
18	Casas M	Univ Illes Balears	Spain	8	42	5.25	4				1	3
19	Markechova D	Constantine Philosopher Univ Nitra	Slovakia	8	22	2.75	2				1	2
20	Chen BD	Xi An Jiao Tong Univ	China	5	101	11.22	6			1	5	7

Abbreviations: TP: total publications; TC: total citations; TC/TP: total citations per publication; *h*: *h*-index; >100, >50, >20, >10, and >5: Number of papers with more than 100, 50, 20, 10, and 5 citations.

**Table 3:** The top-20 most cited documents of the topic from 1999–2018.

Title	Author	Year	Journal	Category	TC	TC/Year	NA	NI	NR
Compressed sensing	Donoho DL	2006	IEEE Transactions on Information Theory	Computer Science	12,392	953.23	1	1	44
Maximum entropy modeling of species geographic distributions	Phillips SJ et al.	2006	Ecological Modeling	Ecology	5798	446.00	3	4	90
The large-N limit of superconformal field theories and supergravity	Maldacena J	1999	International Journal of Theoretical Physics	Physics	5000	250.00	1	1	89
Inference of macromolecular assemblies from crystalline state (review)	Krissinel E et al.	2007	Journal of Molecular Biology	Biochemistry & Molecular Biology	4430	369.17	2	1	119
The cosmological simulation code GADGET-2 (review)	Springel V	2005	Monthly Notices of the Royal Astronomical Society	Astronomy & Astrophysics	3421	244.36	1	1	122
A tutorial on support vector regression (review)	Smola AJ et al.	2004	Statistics and Computing	Computer Science	3216	214.40	2	2	139
Fast and robust fixed-point algorithms for independent component analysis	Hyvärinen A	1999	IEEE Transactions on Neural Networks	Computer Science	3196	159.80	1	1	37
Supercooled liquids and the glass transition (review)	Debenedetti PG et al.	2001	Nature	Multidisciplinary Sciences	2612	145.11	2	2	109
Interactions with aromatic rings in chemical and biological recognition (review)	Meyer EA et al.	2003	Angewandte Chemie International Edition	Chemistry, Multidisciplinary	2563	160.19	3	2	669
Physiological time-series analysis using approximate entropy and sample entropy	Richman JS et al.	2000	American Journal of Physiology-Heart and Circulatory Physiology	Cardiac & Cardiovascular Systems	2441	128.47	2	2	34
Modeling of species distributions with MaxEnt: New extensions and a comprehensive evaluation	Phillips SJ et al.	2008	Ecography	Biodiversity Conservation	2355	214.09	2	2	38
Size-distribution analysis of macromolecules by sedimentation velocity ultracentrifugation and Lamm equation modeling	Schuck P	2000	Biophysical Journal	Biophysics	2339	123.11	1	1	61
Recent developments in magnetocaloric materials (review)	Gschneidner Jr KA et al.	2005	Reports on Progress in Physics	Physics, Multidisciplinary	2077	148.36	3	1	255
Nanostructured high-entropy alloys with multiple principal elements: Novel alloy design concepts and outcomes	Yeh JW et al.	2004	Advanced Engineering Materials	Materials Science, Multidisciplinary	2032	135.47	8	5	26
Survey over image thresholding techniques and quantitative performance evaluation (review)	Sezgin M et al.	2004	Journal of Electronic Imaging	Engineering	1995	133.00	2	2	141
Quantum discord: A measure of the quantumness of correlations	Ollivier H et al.	2002	Physical Review Letters	Physics, Multidisciplinary	1941	114.18	2	1	18
A Rietveld-analysis program RIETAN-98 and its applications to zeolites	Izumi F et al.	2000	European Powder Diffraction, PTS 1 and 2	Materials Science, Multidisciplinary	1901	100.05	2	2	17
A statistical explanation of MaxEnt for ecologists	Elith J et al.	2011	Diversity and Distributions	Biodiversity Conservation	1887	235.88	6	5	58
Transition-metal-based magnetic refrigerants for room-temperature applications	Tegus O et al.	2002	Nature	Multidisciplinary Sciences	1787	105.12	4	1	12
Efficient, multiple-range random walk algorithm to calculate the density of states	Wang FG et al.	2001	Physical Review Letters	Physics, Multidisciplinary	1759	97.72	2	1	29
Average Values		2003			3257	223.88	2.5	1.9	105

Abbreviations: TC: total citations; TC/Year: total citations per year; NA: number of authors; NI: number of institutions; NR: number of references.

**Table 4:** The top-20 most cited documents of the topic from 2014–2018.

Title	Author	Year	Journal	Category	TC	TC/Year	NA	NI	NR
Planck 2013 results. XXII. Constraints on inflation	Planck Collaboration	2014	Astronomy & Astrophysics	Astronomy & Astrophysics	1276	255.20	244	99	333
Microstructures and properties of high-entropy alloys (review)	Zhang Y et al.	2014	Progress in Materials Science	Materials Science, Multidisciplinary	1169	233.80	7	5	297
A fracture-resistant high-entropy alloy for cryogenic applications	Gludovatz B et al.	2014	Science	Multidisciplinary Sciences	871	174.20	6	6	50
First-principles calculations for point defects in solids	Freysoldt C et al.	2014	Reviews of Modern Physics	Physics, Multidisciplinary	646	129.20	7	3	384
Quantifying coherence	Baumgratz T et al.	2014	Physical Review Letters	Physics, Multidisciplinary	574	114.80	3	1	41
A critical review of high entropy alloys and related concepts (review)	Miracle DB et al.	2017	Acta Materialia	Materials Science, Multidisciplinary	551	275.50	2	2	349
Caloric materials near ferroic phase transitions (review)	Moya X et al.	2014	Nature Materials	Chemistry, Physical	487	97.40	3	2	151
G_mmpbsa-A GROMACS tool for high-throughput MM-PBSA calculations	Kumari R et al.	2014	Journal of Chemical Information and Modeling	Chemistry, Medicinal	476	95.20	3	2	102
Rarefaction and extrapolation with hill numbers: A framework for sampling and estimation in species diversity studies	Chao A et al.	2014	Ecological Monographs	Ecology	437	87.40	7	5	70
Cucurbiturils: From synthesis to high-affinity binding and catalysis (review)	Assaf KI et al.	2015	Chemical Society Reviews	Chemistry, Multidisciplinary	429	107.25	2	1	242
The MM/PBSA and MM/GBSA methods to estimate ligand-binding affinities (review)	Genheden S et al.	2015	Expert Opinion on Drug Discovery	Pharmacology & Pharmacy	425	106.25	2	2	105
High-entropy alloys: A critical review (review)	Tsai MH et al.	2014	Materials Research Letters	Materials Science, Multidisciplinary	425	85.00	2	2	143
Metastable high-entropy dual-phase alloys overcome the strength-ductility trade-off	Li ZM et al.	2016	Nature	Multidisciplinary Sciences	420	140.00	5	2	30
The ensemble nature of allostery (review)	Motlagh HN et al.	2014	Nature	Multidisciplinary Sciences	401	80.20	4	1	108
Biomedical applications of supramolecular systems based on host-guest interactions (review)	Ma X et al.	2015	Chemical Reviews	Chemistry, Multidisciplinary	354	88.50	2	1	369
Three dimensional mesoscopic simulation of magnetic field effect on natural convection of nanofluid	Sheikholeslami M et al.	2015	International Journal of Heat and Mass Transfer	Thermodynamics	351	87.75	2	3	25
Entropic stabilization of mixed A-cation ABX (3) metal halide perovskites for high performance perovskite solar cells	Yi CY et al.	2016	Energy & Environmental Science	Chemistry, Multidisciplinary	349	116.33	9	1	33
Self-assembly of colloidal nanocrystals: From intricate structures to functional materials (review)	Boles MA et al.	2016	Chemical Reviews	Chemistry, Multidisciplinary	318	106.00	3	4	748
Black holes and the butterfly effect	Shenker SH et al.	2014	Journal of High Energy Physics	Physics, Particles & Fields	292	58.40	2	2	69
A precipitation-hardened high-entropy alloy with outstanding tensile properties	He JY et al.	2016	Acta Materialia	Materials Science, Multidisciplinary	283	94.33	10	4	44
Average Values		2015			527	126.64	16.3	7.4	185

Abbreviations: TC: total citations; TC/Year: total citations per year; NA: number of authors; NI: number of institutions; NR: number of references.

**Table 5:** The top-20 most cited documents of *Entropy* from 1999–2018.

Title	Author	Year	Special Issues or Subject	Special Issues or Not	TC	TC/Year	NA	NI	NR
Exploration and development of high entropy alloys for structural applications	Miracle DB et al.	2014	High Entropy Alloys	Y	221	44.20	6	1	81
Permutation entropy and its main biomedical and econophysics applications: A review	Zanin M et al.	2012	Concepts of Entropy and Their Applications	Y	215	30.71	4	8	110
Use of maximum entropy modeling in wildlife research (review)	Baldwin RA	2009	Maximum Entropy	Y	210	21.00	1	1	40
Black holes, cosmological solutions, future singularities, and their thermodynamical properties in modified gravity theories	de la Cruz-Dombriz A et al.	2012	Modified Gravity: From Black Holes Entropy to Current Cosmology	Y	158	22.57	2	3	202
Families of alpha-beta-and gamma-divergences: Flexible and robust measures of similarities	Cichocki A et al.	2010	Generalized Divergences	N	128	14.22	2	3	85
Searching for next single-phase high-entropy alloy compositions	Gao MC et al.	2013	High Entropy Alloys	Y	125	20.83	2	2	35
Entropy generation analysis of desalination technologies	Mistry KH et al.	2011	Entropy Generation Minimization	Y	120	15.00	6	2	45
Preclinical diagnosis of magnetic resonance (MR) brain images via discrete wavelet packet transform with Tsallis entropy and generalized eigenvalue proximal support vector machine (GEPSVM)	Zhang YD et al.	2015	Wavelet Entropy: Computation and Applications	Y	117	29.25	5	6	41
Vessel pattern knowledge discovery from AIS data: A framework for anomaly detection and route prediction	Pallotta G et al.	2013	Science and Technology Organization	N	116	19.33	3	1	47
Thermodynamics of thermoelectric phenomena and applications (review)	Goupil C et al.	2011	Local Entropy Production	N	114	14.25	5	4	156
Application of entropy measures on intrinsic mode functions for the automated identification of focal electroencephalogram signals	Sharma R et al.	2015	Entropy and Electroencephalography	Y	104	26.00	3	2	75
Multivariate multi-scale permutation entropy for complexity analysis of Alzheimer’s disease EEG	Morabito FC et al.	2012	Concepts of Entropy and Their Applications	Y	103	14.71	6	3	24
Optimal multi-level thresholding based on maximum Tsallis entropy via an artificial bee colony approach	Zhang YD et al.	2011	Tsallis Entropy	Y	101	12.62	2	1	28
Time series analysis using composite multiscale entropy	Wu SD et al.	2013	Multiscale Entropy	N	99	16.5	5	4	22
Axiomatic characterizations of information measures	Csiszár I	2008	Facets of Entropy-Papers presented at the workshop in Copenhagen	Y	96	8.73	1	1	62
Bearing fault diagnosis based on multiscale permutation entropy and support vector machine	Wu SD et al.	2012	Permutation Entropy	N	92	13.14	5	3	20
On the thermodynamics of friction and wear-a review (review)	Amiri M et al.	2010	Entropy and Friction	Y	89	9.89	2	1	131
Fruit classification by wavelet-entropy and feedforward neural network trained by fitness-scaled chaotic ABC and biogeography-based optimization	Wang S et al.	2015	Machine Learning and Entropy: Discover unknown unknowns in complex data sets	Y	83	20.75	6	4	46
Multicomponent and high entropy alloys (review)	Cantor B	2014	High Entropy Alloys	Y	80	16.00	1	1	45
The multiscale entropy algorithm and its variants: A review (review)	Humeau-Heurtier A	2015	Multiscale Entropy and Its Applications in Medicine and Biology	Y	78	19.50	1	1	44
Average Values		2012			122	19.46	3.4	2.6	66.95

Abbreviations: TC: total citations; TC/Year: total citations per year; NA: number of authors; NI: number of institutions; NR: number of references.

**Table 6:** The details of the topic’s top-11 clusters.

Cluster ID	Proportion	Silhouette	Mean Year of Publications	Label (TF-IDF)	Label (Log-Likelihood Ratio)	Label Terms (Mutual Information)
1	6.72%	0.992	2012	graph entropy	graph entropy	graph entropy
2	6.23%	0.962	2009	Tsallis	relative entropy	conditional Renyi entropy
3	5.41%	0.989	2012	multiscale entropy	permutation entropy	detecting weak abrupt information
4	4.59%	0.951	2001	composability	Clausius entropy	*q*-exponential distribution
6	4.10%	0.928	2007	ads-cft correspondence	entropy function	black hole
7	3.44%	0.995	2005	entropy dimension	local entropy theory	entropy system
8	3.11%	0.987	2013	black holes	entanglement entropy	entanglement entropy
11	2.79%	0.973	2007	residual entropy	order statistics	concomitants of record values
12	2.46%	0.978	2011	Tsallis entropy	pseudo-additive entropy	different entropy formalism
15	1.97%	0.987	2009	sample entropy	ordinal pattern	multiscale permutation
22	1.31%	0.993	2010	maximum entropy	water engineering	dynamical system

**Table 7:** The details of *Entropy*’s top-13 clusters.

Cluster ID	Proportion	Silhouette	Mean Year of Publications	Label (TF-IDF)	Label (Log-Likelihood Ratio)	Label Terms (Mutual Information)
0	8.03%	0.919	2010	fault diagnosis	EEG signal	EEG signal
1	5.42%	0.863	2009	estimating information transfer	information-theoretic perspective	information measures
2	4.96%	0.909	2008	maximum entropy	entropy production principle	MaxEnt formalism
4	3.61%	0.918	2008	nonextensive Tsallis	nonadditive entropy S${}_{q}$	nonadditive entropy
5	2.62%	0.976	2009	genetic algorithm-based identification	genetic algorithm-based identification	parameter identification
6	2.53%	0.950	2008	street network dispersion	statistical distribution	algebraic structures
8	2.17%	0.910	2010	optimization perspective	transfer entropy	transfer entropy
10	1.99%	0.981	2006	dark energy problem	black hole entropy	black holes
11	1.90%	0.969	2007	discrete wavelet entropy	wavelet threshold de-noising	wavelet threshold de-noising
12	1.71%	0.990	2010	irrigation districts	river cross-section	entropy applications
13	1.62%	0.965	2013	complexity measure	complexity measure	non extensive statistical physics
14	1.44%	0.988	2009	entropy method	entropy principle	Volterra polynomials
15	1.35%	1.000	2008	cultural naturalism	cultural naturalism	multiscale entropy analysis

**Table 8:** Main comparisons between the topic and *Entropy*.

Feature	Entropy Topic	*Entropy* Journal
Publication Trend	Owing a tremendous base; at the origination of its exponential increment.	Experiencing an exponential increase with a sharp slope; becoming the most productive entropy-related journal since 2013.
Most Productive Author	Principally working in Asia and North America, especially China, which has 9 authors ranking in the top-20.	Attracting many celebrities of the topic; referring to more diversified institutions and nations.
Most Productive Country	Including the USA, China, Germany, France, etc.; largely located in Europe.	Including China, the USA, Italy, Spain, etc; but enjoying a more extensive geographical distribution.
International Cooperation	Mainly promoted by European countries where Germany and France play a pivotal role.	Enjoying a global cooperation network in which the USA, Italy, Germany, and Saudi Arabia exert major influence.
Most Productive Fund	Countless; primarily related to natural science foundations in Europe, North America, and China.	Similar to the topic’s situation, except that there are more small-sized Chinese funds coming to the fore.
Most Cited Paper	Including many reviews; referring to plenty of disciplines, especially highly related to physics, ecology, computer science, etc.	Relatively new; with good bibliometric indicator performance; mainly published in Special Issues.
Hotspots	Changed significantly during the last 20 years; mainly including three areas at present, i.e., graph entropy, permutation entropy, and pseudo-additive entropy.	Playing a leading role in multiscale entropy and permutation entropy; having several innovative areas, like transfer entropy, discrete wavelet entropy, etc.
